# Functional independence in the Finnish spinal cord injury population

**DOI:** 10.1038/s41393-021-00700-x

**Published:** 2021-09-15

**Authors:** Kirsi Majamäki, Susanna Tallqvist, Aki Vainionpää, Eerika Koskinen, Anna-Maija Kauppila, Paula Bergman, Heidi Anttila, Harri Hämäläinen, Anni Täckman, Mauri Kallinen, Jari Arokoski, Sinikka Hiekkala

**Affiliations:** 1 Faculty of Sport Sciences, University of Jyväskylä, Jyväskylä, Finland; 2 Faculty of Medicine, University of Helsinki, Helsinki, Finland; 3 Department of Rehabilitation, Seinäjoki Central Hospital, Seinäjoki, Finland; 4 Department of Neurosciences and Rehabilitation, Tampere University Hospital, Tampere, Finland; 5 Department of Medical Rehabilitation/Spinal Cord Injury Outpatient Clinic, Oulu University Hospital, Oulu, Finland; 6 Biostatistics Unit, Department of Public Health, University of Helsinki and Helsinki University Hospital, Helsinki, Finland; 7 Finnish Institute for Health and Welfare, Finnish Institute for Health and Welfare (THL), Public Health and Welfare Department, Knowledge Management and Co-creation Unit, Helsinki, Finland; 8 Department of Internal Medicine and Rehabilitation/Spinal Cord Injury Outpatient Clinic, Helsinki University Hospital, Helsinki, Finland; 9 The Finnish Association of Spinal Cord Injured Akson, Helsinki, Finland; 10 Department of Rehabilitation Medicine, Central Finland Health Care District, Central Finland Central Hospital, Jyväskylä, Finland; 11 Center for Life Course Health Research, University of Oulu, Oulu, Finland; 12 The Finnish Association of People with Physical Disabilities, Helsinki, Finland; 13 Validia Rehabilitation, Helsinki, Finland

**Keywords:** Spinal cord diseases, Health care

## Abstract

**Study design:**

A cross-sectional survey of the Finnish population with spinal cord injury (FinSCI database).

**Objectives:**

To describe the functional independence of the population with spinal cord injury (SCI) in Finland and to identify how generic and lesion characteristics affect their functional independence.

**Setting:**

The participants were recruited from the registers of three SCI outpatient clinics responsible for lifelong follow-up and care for people with SCI in Finland.

**Methods:**

The data were retrieved from FinSCI (*n* = 1772). The response rate was 50% (*n* = 884). The Spinal Cord Independence Measure-Self Report (SCIM-SR) was used. The data were analyzed with univariate testing, factor analyses, and multiple linear regression models.

**Results:**

The median (percentiles 25; 75) SCIM-SR total score was 76.0 (58.8; 89.0), and the score was 18.0 (13.0; 20:0) for the self-care sub-scale, 33.0 (25.0; 39.0) for the respiration and sphincter management sub-scale and 29.0 (16.0; 36.8) for the mobility sub-scale. The higher the neurological level in groups AIS A, B, and C, the lower the functional ability. Group AIS D at any injury level had the highest level of functional ability. Age and the number of years since injury negatively influenced the SCIM-SR scores for every sub-scale.

**Conclusion:**

Based on the International Spinal Cord Injury Core Data Set, the severity of SCI can differentiate persons with SCI according to their functional ability. The results suggest that SCI affects individuals’ health more than ageing alone does, thereby reducing the functional ability and independence of persons with SCI over time.

## Introduction

Spinal cord injury (SCI) can cause sensory and motor loss and changes in the autonomic nervous system [1]. Tetraplegia, a cervical SCI, affects the sensory and motor function of the arms, body, and legs. Individuals with lesions at the C4 or higher may need ventilation assistance. Paraplegia, a thoracic or lumbar SCI, impacts the function of the trunk and legs [2]. Neurological status is the strongest predictor of functional independence [3]. Other factors such as secondary health conditions, psychological, social, and environmental supports, and cognitive ability can also influence the outcomes. For individuals with a complete SCI, the optimal level of functional independence can be estimated using outcome-based practice guidelines. For persons with incomplete SCI, the goal-setting process for the function is more individualised [4].

The International Standards for Neurological Classification of SCI (ISNCSCI) are used to assess the neurological level and completeness of SCI. The International SCI Core Data Set provides recommendations for the standardisation of reporting, and it is recommended that the severity of SCI be grouped by ISNCSCI [5]. The purpose of the International SCI Core Data Set is to give SCI studies a standardised way of collecting and reporting data, which would enable the results of one SCI study to be compared with those of another [6].

As part of the Finnish Spinal Cord Injury Study (FinSCI) [7], this study aimed to describe the functional independence of a population with SCI in Finland by using the spinal cord independence measure-self report (SCIM-SR) [8]. It also examined how generic and lesion characteristics, classified by the recommendations of the International SCI core Data set, affected SCIM-SR scores [5].

## Methods

### Design

FinSCI is a collaborative study among The Finnish Association of People with Physical Disabilities, The Finnish Association of Spinal Cord Injured Akson, The Finnish Institute of Health and Welfare, and the SCI outpatient clinics of three university hospitals (Oulu, Tampere and Helsinki). These three university hospitals are responsible for acute care, immediate rehabilitation and life-long multi-professional follow-up, care and rehabilitation of persons with SCI in all of Finland. The purpose of FinSCI is to identify factors related to the health and functioning of persons with SCI, their challenges with accessibility, and how such factors are interconnected [7].

### Sample

Study participants were recruited from the registers of the SCI outpatient clinics from the university hospitals in Oulu, Tampere and Helsinki. Their clinical data, consisting of general and lesion characteristics including ISNCSCI [5] to determine The American spinal injury association impairment scale classification (AIS) and the neurological level of the injury, were also collected from these registers. Inclusion criteria were as follows: age of at least 16 years; non-traumatic SCI (NTSCI) or traumatic SCI (TSCI); and AIS of A, B, C or D. Patients with AIS E, living in an institute or with a congenital SCI, progressive or neurodegenerative disease, multiple sclerosis, amyotrophic lateral sclerosis or Guillain-Barre syndrome were excluded. The detailed protocol, the precise patient selection process and the content of the formulated questionnaire have been presented elsewhere [7]. The questionnaire was sent to the participants in February 2019, and the answers were collected until the end of July 2019.

### Outcome measure

This study evaluates the functioning of the Finnish population with SCI using the SCIM-SR [8], which was based on the clinician-administered SCIM III (spinal cord independence measure version III), an internationally used measure for the functional assessment of SCI populations [9]. The first version of SCIM was published in 1997 and was used as a multidisciplinary instrument to assess functioning in people with SCI [10].

SCIM-SR is a self-report outcome measure that is commonly used in community-based settings [11]. Self-reported indicators are easy to use and are targeted for a focus group [12]. Self-reports take less time [12, 13], require fewer resources [12] and are a cost-effective way of measuring functional ability [13]. They are valid for the evaluation of self-care functioning in populations with disabilities and can be used in inpatient and outpatient situations [12]. The results of SCIM-SR are correlated with those of the SCIM III [8, 14–16], which has good validity and reliability [8]. SCIM-SR has been used in a national study in Switzerland (SwiSCI) [17] and in the international survey InSCI [18]. The use of SCIM-SR has been recommended in outpatient and community settings, as well as in acute and post-acute rehabilitation settings [8]. It is useful for monitoring changes in functional independence [19]. SCIM-SR covers 12 of the 43 preselected categories from the International Classification of Functioning, Disability and Health (ICF) [20] from the FinSCI dataset [7].

SCIM-SR consists of 17 items divided into three sub-scales: self-care, respiration and sphincter management, and mobility. For each item, the person evaluates their present situation and their need for assistance to complete the activities. Self-care (score range 0–20) includes six items related to eating and drinking, washing, dressing and grooming. There were 2 items addressing upper body and lower body functions. Respiration and sphincter management (score range 0–40) were covered by four items, with questions about breathing, bladder and bowel management, and toilet use; three items addressed bladder and bowel management. There are nine items on mobility (score range 0–40), which address the need for assistance and the ability to move around. The SCIM-SR total score ranges between 0 and 100. The higher the score, the better the individual’s level of independent functioning [8].

### Statistical analyses

Descriptive statistics were used to summarise the characteristics of the study population. The data were not normally distributed, so nonparametric tests were used. The SCI-related demographics are presented as means (and standard deviations); frequencies (and percentages); or medians (and 25% and 75% percentiles), depending on the distribution of the data. Factor analyses were used to explore SCIM-SR’s ability to distinguish groups differing in SCI severity. Differences among the groups were examined with nonparametric tests (Kruskal–Wallis, Mann–Whitney *U* test, Wilcoxon). The significance values for pairwise comparisons were adjusted by Bonferroni correction for multiple tests. In addition, multiple linear regression models were used to study how age, SCI severity and the number of years since injury are associated with the total score. *P*-values <0.05 were considered statistically significant. Statistical analyses were carried out using SPSS version 24 software (SPSS Inc., Chicago, Illinois, USA). There were some missing values in the dataset (self-care 4.4%, respiration and sphincter management 15.6%, mobility 11.5%). The number of missing values did not differ between the SCI severity groups. The missing values were replaced with the medians for all sub-scales, and they were calculated separately for each SCI severity group.

## Results

### Characteristics of participants and non-respondents

There was a statistically significant difference between the participants (*n* = 884) and the non-responders (*n* = 888) in gender and age (Table 1). Younger persons answered less frequently, and persons aged 61–75 and females participated actively.

**Table 1:** Eligible population of the FinSCI study divided into participants (N884) and non-respondents (N888).

	Participants N884	Non-respondents N888	*p* value
	*n* (%)	*n* (%)	
Gender			<0.01
Female	307 (35%)	253 (29%)	
Male	577 (65%)	633 (71%)	
Age, years	(min 20, max 90, mean 61, SD 14)	(min 17, max 93, mean 54, SD 17)	<0.01
	median 63 IQR 53–71	median 55 IQR 40–68	
20–30	34 (4%)	96 (11%)	
31–45	108 (12%)	204 (23%)	
46–60	238 (27%)	243 (27 %)	
61–75	386 (44%)	243 (27%)	
≥76	118 (13%)	102 (12%)	
Aetiology			0.1
Traumatic	492 (56%)	527 (59%)	
Non-traumatic	392 (44%)	361 (41%)	
Severity of SCI			0.21
C1–4 AIS A, B, and C	95 (12%)	107 (11%)	
C5–8 AIS A, B, and C	55 (6%)	62 (7%)	
T1–S5 AIS A, B, and C	184 (21%)	209 (24%)	
AIS D at any injury level	550 (62%)	510 (57%)	
Years since injury	(min 1, max 67, mean 11, SD 11)	(min 1, max 66, mean 10, SD 10)	0.52
	median 7 IQR 4–14	median 6 IQR 4–14	
1–5 years	353 (40%)	379 (43%)	
6–10 years	227 (26%)	222 (25%)	
11–15 years	128 (14%)	111 (12%)	
≥16 years	176 (20%)	176 (20%)	

The youngest participant was 20 years old, which is why the first age group is 20–30 years. The oldest participant was 90 years of age. Two participants used a ventilator and were included in groups C1–4AIS A, B and C based on the recommendations of the International Spinal Cord Injury Core Data Set [5]. There was one transgender participant who was grouped by gender according to the hospital records (Table 1). The shortest time since injury was 11 months, and this value was rounded up to one year. There were some statistically significant differences in the distributions of generic and lesion characteristics among the participants, which are presented in the Supplement (Supplementary Table A).

**Fig. 1:**
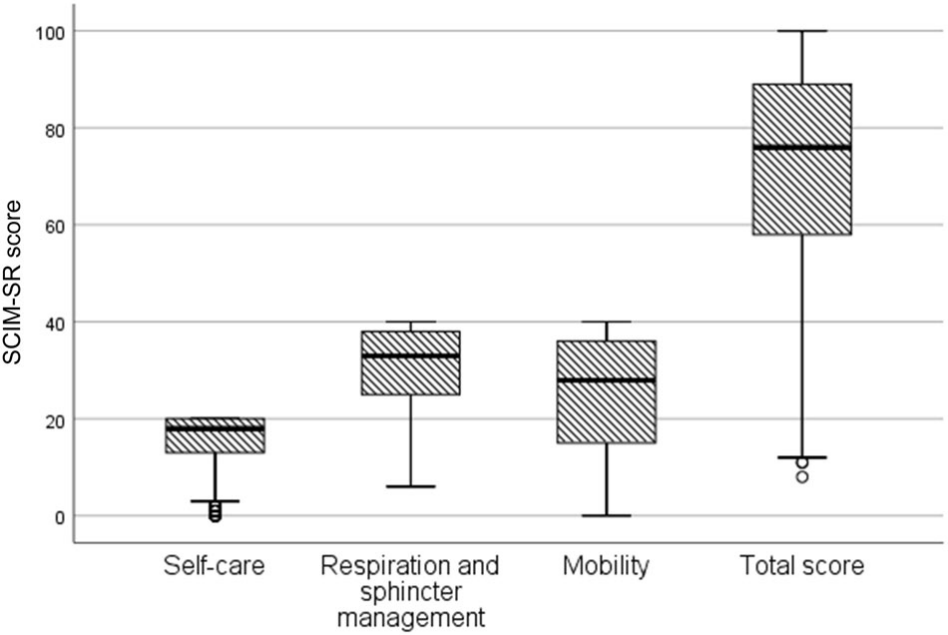
The sub-scale and total scores of the SCIM-SR for the population with SCI in the FinSCI study (*n* = 884) (median, percentiles 25; 75). Self-care sub-scale observed range 0–20; respiration and sphincter management sub-scale observed range 6–40; mobility sub-scale observed range 0–40; total score observed range 8–100.

Regarding self-care, 292 (34%) of the respondents received the maximum score; regarding respiration and sphincter management, 176 (21%) received the maximum score; for mobility, 162 (18%) received the maximum score; and 77 (10%) received the maximum total score, respectively (Fig. 1).

### Total score

Gender, age, the severity of SCI, aetiology and the number of years since injury had statistically significant effects on the SCIM-SR total score (Table 2). The females had higher scores than the males. The scores decreased with the number of years since injury, and there was a statistically significant difference between all the years since injury groups in the pairwise comparisons. The 46–60 age group had the highest score, and the 31–45 age group scored lowest. There was a statistically significant difference between the groups aged 31–45 and 46–60 and between those aged 46–60 and 61–75. AIS D at any injury level (later referred to as group AIS D) scored highest, and group C1–4 AIS A, B and C had the lowest scores. In the pairwise comparisons of the SCI severity groups, there were significant differences between group AIS D and all the other SCI severity groups and between groups C1–4 AIS A, B and C and T1–S5 AIS A, B and C. The NTSCI group had higher scores than did the TSCI group.

**Table 2:** Crosstabulation of gender, age in years, the severity of SCI, aetiology, years since injury and SCIM-SR sub-scales self-care, respiration and sphincter management, mobility and total score in the Finnish spinal cord injury (FinSCI) survey.

		Self-care	Respiration and sphincter management	Mobility	Total score
	Scale range	0–20	0–40	0–40	0–100
	Observed range	0–20	6–40	0–40	8–100
Gender	Female	18.0 (14.8, 20.0)	33.0 (27.0, 39.0)	30.0 (17.0, 37.0)	80.0 (64.8, 90.0)
	Male	18.0 (12.0, 20.0)	33.0 (25.0, 38.0)	27.0 (15.0, 36.0)	73.5 (55.0, 87.8)
	*p*	0.076	0.313	0.073	0.011
Age, years	20–30	18.0 (16.0, 20.0)	33.0 (25.0, 37.0)	20.5 (14.0, 38.5)	73.0 (57.0, 91.0)
	31–45	18.0 (16.0, 20.0)	31.0 (26.0, 36.0)	19.0 (16.0, 34.8)	70.0 (59.0, 87.0)
	46–60	18.0 (15.0, 20.0)	33.0 (27.0, 39.0)	33.0 (18.0, 40.0)	81.0 (66.5, 94.0)
	61–75	17.0 (11.0, 20.0)	33.0 (25.0, 39.0)	29.0 (14.0, 35.0)	75.5 (54.5, 88.0)
	≥76	16.0 (10.0, 18.0)	33.0 (22.8, 39.0)	27.5 (15.8, 34.0)	77.0 (57.0, 87.0)
	*p*	<0.001	0.406	<0.001	0.011
Severity of SCI	C1–4 AIS A, B, and C	5.5 (1.0, 13.0)	21.0 (17.0, 26.0)	8.0 (3.0, 14.0)	36.5 (25.0, 49.0)
	C5–8 AIS A, B, and C	14.0 (8.0, 18.0)	25.0 (21.0, 28.0)	13.5 (9.0, 17.0)	54.5 (47.0, 59.0)
	T1–S5 AIS A, B, and C	18.0 (16.0, 19.0)	31.0 (28.0, 34.0)	17.0 (14.0, 19.0)	67.0 (60.0, 71.0)
	AIS D at any injury level	19.0 (15.0, 20.0)	36.0 (30.0, 40.0)	34.0 (28.0, 40.0)	87.0 (78.0, 95.0)
	*p*	<0.001	<0.001	<0.001	<0.001
Aetiology	Traumatic	18.0 (11.0, 20.0)	31.0 (25.0, 36.0)	20.0 (14.0, 34.0)	69.0 (54.5, 87.0)
	Non-traumatic	18.0 (14.5, 20.0)	35.0 (29.0, 40.0)	33.0 (19.0, 39.0)	84.0 (67.0, 92.8)
	*p*	<0.001	<0.001	<0.001	<0.001
Years since injury	1–5 years	18.0 (14.0, 20.0)	36.0 (29.0, 40.0)	34.0 (25.5, 40.0)	87.0 (69.0, 95.5)
	6–10 years	18.0 (13.0, 20.0)	33.0 (26.0, 39.0)	30.0 (15.0, 39.0)	81.0 (59.0, 90.5)
	11–15 years	18.0 (13.0, 20.0)	31.0 (25.0, 35.0)	19.0 (15.0, 34.0)	69.0 (56.0, 86.0)
	≥16 years	17.0 (11.0, 18.0)	31.0 (23.0, 33.0)	16.0 (10.0, 33.0)	65.0 (14.0, 73.0)
	*p*	<0.001	<0.001	<0.001	<0.001

All scores are expressed as median with (25% and 75% percentiles).

The SCIM-SR scores can distinguish the SCI severity groups. AIS D and T1–S5 AIS A, B and C groups were clearly distinguished from the other groups, but groups C1–4 AIS A, B and C and C5–8AIS A, B and C seemed more difficult to separate from one another (Supplement; Supplementary statistical analyses, Supplementary Table B, and Supplementary Fig. A).

### Self-care sub-scale

Ageing decreased individuals’ ability to perform self-care, as shown by the pairwise comparisons between the groups aged 46–60 years and 61–75 years and between those 76 years or older and all other age groups (Table 2). The severity of SCI was a significant factor influencing functioning. Group AIS D had the highest scores, and group C1–4AIS A, B and C had the lowest. The scores for functioning were significantly different in the pairwise comparisons between all SCI severity groups. There was a statistically significant difference between the TSCI and NTSCI groups, with the NTSCI group having higher scores. The persons who had been injured 5 or fewer years prior had the highest self-care scores, and those who had been injured 16 or more years prior had the lowest self-care scores.

### Respiration and sphincter management sub-scale

The respiration and sphincter management sub-scale scores differed statistically significantly according to all the lesion characteristics but not the generic characteristics (Table 2). Furthermore, in the pairwise analyses of the SCI severity groups, there was a statistically significant difference between all the groups except between group C1–4 AIS A, B and C and group C5–8AIS A, B and C. Group AIS D had the highest scores, and group C1–4 AIS A, B and C had the lowest. The TSCI group had statistically significantly lower scores than did the NTSCI group. The participants who had been injured 5 or fewer years ago had the highest scores, and those who had been injured 11 or more years ago had the lowest scores. In the pairwise analyses, the difference was statistically significant between all the years since injury groups.

### Mobility sub-scale

The 31–45 age group had the lowest mobility scores, and the 46–60 age group had the highest (Table 2). In the pairwise comparisons of the age groups, there was a statistically significant difference between age groups 31–45 and 46–60, 46–60 and 61–75, and 46–60 and ≥76. The severity of SCI was a notable factor influencing mobility. Group AIS D had the highest scores, and group C1–4 AIS A, B and C had the lowest scores. Group AIS D differed significantly from all other SCI severity groups, and in addition, there was a statistically significant difference between group C1–4 AIS A, B and C and group T1–S5 AIS A, B and C. The NTSCI group had higher mobility scores than did the TSCI group. The participants who had been injured 5 or fewer years prior had the highest scores, and those injured 16 or more years prior had the lowest scores. The scores tended to decrease as the time since injury increased. As with the respiration and sphincter management sub-scale, there was a statistically significant difference between the years since injury groups.

### Interrelations of age and years since the injury with the SCI severity groups

**Fig. 2:**
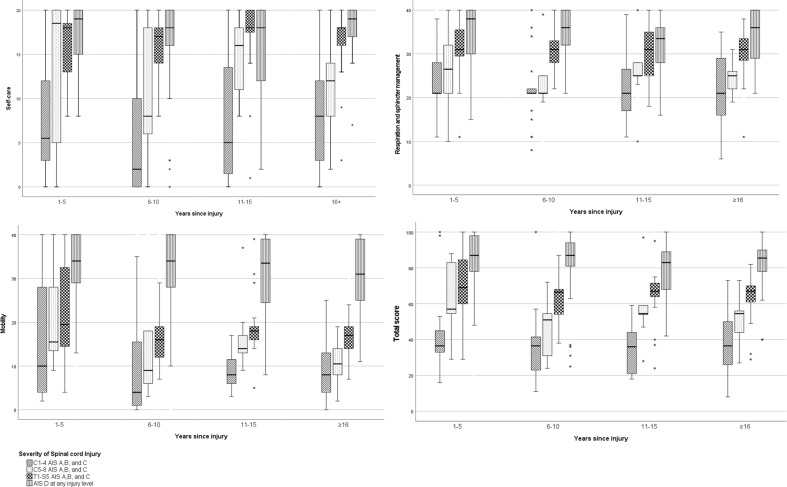
Distribution of the severity of SCI across the years since injury groups in terms of the SCIM-SR sub-scales (self-care observed range 0–20, respiration and sphincter management observed range 6–40, mobility observed range 0–40 and total score observed range 8–100) (median, percentiles 25; 75).

The effect of time since injury in years on the SCIM-SR scores was analysed separately within each SCI severity group (Fig. 2). For the self-care sub-scale, there was a statistically significant difference within group T1–S5 AIS A, B and C between the groups with 6–10 and 11–15 years since the injury. For the respiration and sphincter management sub-scale, there were significant differences within group AIS D in the pairwise comparisons between groups 1–5 and 11–5 years since injury, and between the 6–10 and 11–15 years since injury groups. For the mobility sub-scale, there was a statistically significant difference within group C5–8 AIS A, B and C in the pairwise comparisons between the groups with 1–5 and 16 or more years since the injury. In addition, for the total scores of group AIS D, there was a statistically significant difference between the groups with 1–5 and 11–15 years since the injury. Despite some statistically significant differences, there was no consistency in the results (Fig. 2).

Multiple linear regression was run to understand the effects of age, the severity of SCI and the number of years since the injury on the SCIM-SR total score (Table 3). The levels of homoscedasticity and normality of the residuals were satisfactory, as assessed by visual inspection, and no significant outliers were present, as assessed by Cook’s distance. The model was statistically significant F (7.802) = 111.52; *p* < 0.001 and accounted for 49% of the variation in the SCIM-SR total score. Older age was associated with a lower SCIM-SR total score. Groups C1–4 AIS A, B and C, C5–8 AIS A, B and C, and T1–S5 AIS A, B and C were associated with lower SCIM-SR total scores than group AIS D. The 1–5 years since injury group had a higher SCIM-SR total score than did the ≥16 years since injury group. The other years since injury groups did not differ statistically significantly from the ≥16 years since injury group.

**Table 3:** Results of the linear regression analysis concerning the associations between SCIM-SR total scores and variables of age, severity of SCI groups (C1–4 A, B and C; C5–8 A, B and C; T1–S5 A, B and C; Group D) and years since injury groups (1–5; 6–10; 11–15; ≥16) in the FinSCI study.

		Regression coefficient (95 % CI)	*p*-value
SCIM-SR Total score	Intercept	94.8 (88.9; 100.7)	<0.001
	Age	–0.2 (–0.3; –0.1)	<0.001
	C1–4 AIS A, B, and C	–43.2 (–46.9; –39.5)	<0.001
	C5–8 AIS A, B, and C	–29.1 (–33.7; –24.5)	<0.001
	T1–S5 AIS A, B, and C	–17.9 (–20.8; –14.9)	<0.001
	AIS D at any injury level	reference	
	1–5 years since injury	3.4 (0.1; 6.7)	0.045
	6–10 years since injury	1.4 (–2.0; 4.7)	0.428
	11–15 years since injury	–1.5 (–5.3; 2.3)	0.436
	≥16 years since injury	Reference	
Adjusted *R*^2^: 0.49			

## Discussion

This study used SCIM-SR to assess functional independence among the Finnish population with SCI. All generic and lesion characteristics had some impact on the scores, but the aetiology, the severity of SCI and the number of years since injury affected all sub-scores and total scores. Persons with NTSCI had a higher level of functional independence than persons with TSCI. Those with the most severe SCI (C1–4 AIS A, B and C) had the most limitations. In the group with ISNCSCI AIS A, B and C, the scores increased as the level of SCI decreased. Group AIS D had the highest level of functional ability. The time since injury negatively impacted the scores since the persons who were injured the earliest had the lowest level of functional independence. In addition, older persons tend to have lower total scores.

In the mobility sub-scale and total score, the two youngest age groups had the lowest scores. This contrast to our main results can be explained by the smaller group size and the higher incidence of TSCI in the youngest age groups. The missing values were replaced with the medians, and because of its nature, median imputation can introduce some bias into the results. This aspect should be noted when interpreting the results. The ceiling effect, that is, the high proportion of observations with a maximum score was considerable in the sub-scales of self-care and in respiration and sphincter management. This was mainly related to the overall good function of the participants, and to the large representative sample of persons with NTSCI in group AIS D.

Currently, two studies using SCIM-SR have been published [19, 21]. Although the analysing methods of these studies differ from ours, Prodinger et al. [19] indicated that the generic and lesion characteristics are relevant factors in the analyses of SCIM-SR results, and we agree with that. Based on the results of InSCI [21], persons with complete tetraplegia had more problems with their functional independence than persons with paraplegia or incomplete tetraplegia, as in our study. In contrast to our results, the time since injury did not have a similar kind of negative impact on functional independence; for example, dressing lower body and grooming (parts of the self-care sub-scale) and moving <100 m (part of the mobility sub-scale) were more problematic among the participants who had been injured 5 or fewer years prior in comparison to those who have been injured for a longer time [21]. The fact that the Finnish population with SCI was older than the population in InSCI might partly explain this result.

Clinician-administered SCIM III has been used in several studies, providing valuable information on functional independence among the population with SCI [22–28]. Unfortunately, neither SCIM III nor the self-reported SCIM (SCIM-SR) based on SCIM III has thresholds or reference values, and they are not validated for persons with NTSCI. Therefore, a more specific evaluation of the level of functional independence in the Finnish population with SCI is not possible.

### Strengths and limitations

One of the strengths of this study is that the participants, along with their generic and lesion characteristics, were collected from the registers of SCI outpatient clinics. This made it possible to find the majority of persons with SCI in Finland and combine their characteristics reliably. Persons with SCI actively participated in planning and performing the FinSCI study, which can be seen as an undisputed asset. The response rate was only 50%; however, this is satisfying in comparison to response rates varying from 23% to 54% in countries with defined sampling frames in InSCI [29].

Since the clinical examinations of the participants were performed by several different doctors and physiotherapists between 2000 and 2018, we recognise that the validity and reliability of the ISNCSCI results in our study may be questioned. In addition, ISNCSCI is not fully validated to measure persons with NTSCI. However, the most recent data found in the medical records was used, and persons with progressive or neurodegenerative diseases were excluded.

Analyses of the participants and non-respondents indicate that younger individuals with SCI were not extremely interested in responding to the survey, which might have altered the overall division of the participants according to SCI severity. The high incidence of individuals in SCI severity group AIS D was expected since ~65% of the Finnish SCI population belongs to this group [30]. Largely because of this factor, some of the data are skewed, and the results must be interpreted with caution. Group AIS D differed significantly from the other SCI severity groups since it included more elderly persons, persons with NTSCI and persons who were injured fewer than 5 years prior. Additionally, self-reporting can be seen as doubtful. Participation of persons with the most severe SCI might be affected by the need for assistance in answering. The evaluation of sphincter management was challenging and can reflect the higher number of non-responding participants to that specific area of questioning.

## Conclusions and future directions

Our results suggest that SCI consumes more health than ageing alone does. The time since injury had a negative impact on functional independence as early as 6–10 years after SCI, and the scores decreased between all the time-since-injury groups. This result is worrying and seems to differ from the international results [21]. Recognising this problem can help us plan for future needs regarding health care and rehabilitation for persons with SCI. In particular, the needs of elderly persons with SCI should be carefully evaluated and supported.

This study provided baseline information on the functional ability of the Finnish population with SCI. Although SCIM-SR does not involve psychosocial components that are relevant for everyday life, it evaluates a person’s ability to manage their daily routines. These results can be used as reference values in the future. The FinSCI project itself promoted SCIM-SR for use in SCI outpatient clinics, and we believe that SCIM-SR will be widely used in Finland in the future.

The third conclusion of this study is that, although the ISNCSCI is not a functional measure, we think that grouping persons by SCI severity based on the ISNCSCI and the standardisation of reporting by the International SCI Core Data Set [5] are well suited for the SCIM-SR analyses. SCIM-SR should be further developed to account for these standardisations. In addition, when measuring persons with good functional independence (as group AIS D in our study), it would be desirable if SCIM-SR could describe the possible differences in functioning in even more detail and avoid the possible ceiling effect.

It would be useful if future research could determine internationally evaluated reference values for SCIM-SR. In addition, analyses of personal changes in functional ability that occur over time would provide the most useful information. This detailed information may help both persons with SCI and professionals working with them to estimate levels of functional independence and set realistic goals for rehabilitation to improve the daily lives of persons with SCI.

## Supplementary information

Supplement

## Data Availability

The authors will consider any reasonable requests to access the data.

## References

[1] Kirshblum SC, Burns SP, Biering-Sorensen F, Donovan W, Graves DE, Jha A (2011). International standards for neurological classification of spinal cord injury (revised 2011). J Spinal Cord Med.

[2] Bickenbach J, Boldt I, Brinkhof M, Chamberlain J, Cripps R, Fitzharris M, Bickenbach J, Officer A, Shakespeare T, von Groote P (2013). A global picture of spinal cord injury. International perspectives on spinal cord injury.

[3] Middleton JW, Truman G, Geraghty TJ (1998). Neurological level effect on the discharge functional status of spinal cord injured persons after rehabilitation. Arch Phys Med Rehabil.

[4] Consortium for Spinal Cord Medicine. Outcomes following traumatic spinal cord injury: clinical practice guidelines for health-care professionals. Paralyzed Veterans of America; 1999. 10.1080/10790268.2000.11753539 17536300

[5] Biering-Sorensen F, DeVivo MJ, Charlifue S, Chen Y, New PW, Noonan V (2017). International spinal cord injury core data set (version 2.0)-including standardization of reporting. Spinal Cord.

[6] Devivo MJ (2012). Epidemiology of traumatic spinal cord injury: trends and future implications. Spinal Cord.

[7] Tallqvist S, Anttila H, Kallinen M, Koskinen E, Hämäläinen H, Kauppila A-M (2019). Health, functioning and accessibility among spinal cord injury population in Finland: protocol for the FinSCI study. J Rehabil Med.

[8] Fekete C, Eriks-Hoogland I, Baumberger M, Catz A, Itzkovich M, Luthi H (2013). Development and validation of a self-report version of the spinal cord independence measure (SCIM III). Spinal Cord.

[9] Itzkovich M, Shefler H, Front L, Gur-Pollack R, Elkayam K, Bluvshtein V (2018). SCIM III (spinal cord independence measure version III): reliability of assessment by interview and comparison with assessment by observation. Spinal Cord.

[10] Catz A, Itzkovich M, Agranov E, Ring H, Tamir A, SCIM - (1997). Spinal cord independence measure: a new disability scale for patients with spinal cord lesions. Spinal Cord.

[11] Dorevitch M (1988). The ‘questionnaire’ versus the ‘direct observation’ approach to functional assessment. Br J Rheumatol.

[12] Hoenig H, Hoff J, McIntyre L, Branch LG (2001). The self-reported functional measure: predictive validity for health care utilization in multiple sclerosis and spinal cord injury. Arch Phys Med Rehabil.

[13] Hoenig H, McIntyre L, Sloane R, Branch LG, Truncali A, Horner RD (1998). The reliability of a self-reported measure of disease, impairment, and function in persons with spinal cord dysfunction. Arch Phys Med Rehabil.

[14] Michailidou C, Marston L, De Souza LH (2016). Translation into Greek and initial validity and reliability testing of a modified version of the SCIM III, in both English and Greek, for self-use. Disabil Rehabil.

[15] Aguilar-Rodríguez M, Peña-Pachés L, Grao-Castellote C, Torralba-Collados F, Hervás-Marín D, Giner-Pascual M (2015). Adaptation and validation of the Spanish self-report version of the Spinal Cord Independence Measure (SCIM III). Spinal Cord.

[16] Bonavita J, Torre M, China S, Bressi F, Bonatti E, Capirossi R (2016). Validation of the Italian version of the spinal cord independence measure (SCIM III) self-report. Spinal Cord.

[17] Brinkhof MWG, Fekete C, Chamberlain JD, Post MWM, Gemperli A, SwiSCI Study Group. (2016). Swiss national community survey on functioning after spinal cord injury: protocol, characteristics of participants and determinants of non-response. J Rehabil Med.

[18] Gross-Hemmi MH, Post MW, Ehrmann C, Fekete C, Hasnan N, Middleton JW (2017). Study protocol of the international spinal cord injury (InSCI) community survey. Am J Phys Med Rehabil.

[19] Prodinger B, Ballert CS, Brinkhof MW, Tennant A, Post MW (2016). Metric properties of the spinal cord independence measure—self report in a community survey. J Rehabil Med.

[20] WHO. (2001). International classification of functioning, disability and health (ICF).

[21] Ehrmann C, Reinhardt JD, Joseph C, Hasnan N, Perrouin-Verbe B, Tederko P (2020). Describing functioning in people living with spinal cord injury across 22 countries: a graphical modeling approach. Arch Phys Med Rehabil.

[22] Osterthun R, Tjalma TA, Spijkerman DCM, Faber WXM, van Asbeck FWA, Adriaansen JJE (2020). Functional independence of persons with long-standing motor complete spinal cord injury in the Netherlands. J Spinal Cord Med.

[23] Jörgensen S, Iwarsson S, Lexell J (2017). Secondary health conditions, activity limitations, and life satisfaction in older adults with long-term spinal cord injury. PMR.

[24] Cowan RE, Anderson KD (2019). Replication and novel analysis of age and sex effects on the neurologic and functional value of each spinal segment in the US healthcare setting. Spinal Cord.

[25] Kaminski L, Cordemans V, Cernat E, M’Bra KI, Mac-Thiong JM (2017). Functional outcome prediction after traumatic spinal cord injury based on acute clinical factors. J Neurotrauma.

[26] Tomioka Y, Uemura O, Ishii R, Liu M (2019). Using a logarithmic model to predict functional independence after spinal cord injury: a retrospective study. Spinal Cord.

[27] Ariji Y, Hayashi T, Ideta R, Koga R, Murai S, Towatari F (2020). A prediction model of functional outcome at 6 months using clinical findings of a person with traumatic spinal cord injury at 1 month after injury. Spinal Cord.

[28] Corallo V, Torre M, Ferrara G, Guerra F, Nicosia G, Romanelli E (2017). What do spinal cord injury patients think of their improvement? A study of the minimal clinically important difference of the spinal cord independence measure III. Eur J Phys Rehabil Med.

[29] Fekete C, Brach M, Ehrmann C, Post MWM, InSCI, Stucki G (2020). Cohort profile of the international spinal cord injury community survey implemented in 22 countries. Arch Phys Med Rehabil.

[30] Koskinen E, Väärälä E, Alen M, Kallinen M, Vainionpää A (2017). Selkäydinvammojen ilmaantuvuus on ennakoitua suurempi [Incidence of spinal cord injuries in Finland higher than expected. Lääkärilehti.

