# Supplementary material for: Functional independence in the Finnish spinal cord injury population
Source: Spinal Cord. 2021 Sep 15;60(7):628–34. doi: 10.1038/s41393-021-00700-x (PMC9287165; doi:10.1038/s41393-021-00700-x)

Kirsi Majamäki, PT, Susanna Tallqvist, MSc; PhD; Aki Vainionpää, MD, PhD; Eerika Koskinen, MD, Anna-Maija Kauppila MD PhD; Paula Bergman, MSc; Heidi Anttila, PhD; Harri Hämäläinen, MD, PhD; Anni Täckman, BBA; Mauri Kallinen, MD, PhD; Jari Arokoski, MD, PhD; and Sinikka Hiekkala, PhD

Functional independence in the Finnish spinal cord injury population

Spinal Cord, 2021

Legends:

Supplementary Table A. Internal analyses of the participants of the FinSCI study, N884

Supplementary statistical analyses

Supplementary Table B. Factor loadings of the SCIM-SR in the FinSCI study.

Supplement Figure A. Distributions of the factor scores across severity of SCI groups (median, percentiles 25; 75, min, max) in the FinSCI study.

Supplementary Table A. Internal analyses of the participants of the FinSCI study, N884

		Severity of SCI				
		C1-4 AIS A, B, and C	C5-8 AIS A, B, and C	T1-S5 AIS A, B, and C	AIS D at any injury level	p value
Gender		n (%)	n (%)	n (%)	n (%)	<0.01
	Female	16 (17%)	12 (22%)	61 (33%)	218 (40%)	
	Male	79 (83%)	43 (78%)	123 (67%)	332 (60%)	
Age groups						< 0.01
	20-30	5 (5%)	5 (9%)	10 (5%)	14 (2%)	
	31-45	14 (15%)	13 (24%)	43 (23%)	47 (7%)	
	46-60	21 (22%)	12 (22%)	47 (26%)	158 (29%)	
	61-75	47 (50%)	20 (36%)	73 (40%)	246 (45%)	
	≥ 76	8 (8%)	5 (9%)	11 (6%)	94 (17%)	
Years since SCI						<0.01
	1-5 years	21 (22%)	10 (18%)	28 (15%)	294 (53%)	
	6-10 years	23 (24%)	9 (16%)	46 (25%)	149 (27%)	
	11-15 years	11 (12%)	14 (26%)	39 (21%)	64 (12%)	
	≥ 16	40 (42%)	22 (40%)	71 (39%)	43 (8%)	
Aetiology						<0.01
	Traumatic	83 (87%)	45 (82%)	127 (69%)	237 (43%)	
	Non- traumatic	12 (13%)	10 (18%)	57 (31%)	313 (57%)	

Supplementary statistical analyses

Factor analysis (FA) was conducted on the data for a 19-item SCIM-SR questionnaire that measured self-care, respiration and sphincter management, and mobility to explore how well the questionnaire distinguishes between the four SCI severity groups. The PAF extraction method was used. The overall Kaiser-Meyer-Olkin (KMO) measure was 0.95, which is considered ‘meritorious’ according to Kaiser (1). Bartlett's test of sphericity was statistically significant ($p < .001$), indicating the data were factorizable.

FA revealed two components that had eigenvalues > 1 and that explained 57.9% and 11.0% of the total variance, respectively (69.9% of the total variance). A Varimax orthogonal rotation was employed for interpretability. The factor loadings are presented in Supplementary Table 2. The variables with loads for Factor 1 were related to daily activities, and the variables with loads on Factor 2 were related to mobility. Thus, the method of data interpretation is consistent with the original purpose of the questionnaire: Factor 1, named ‘Daily activities’, covers self-care, respiration and sphincter management, and Factor 2, named ‘Mobility’, covers mobility.

In addition, the factor scores were saved as variables in the dataset, and Kruskal-Wallis tests were used to study the possible differences in factor scores between SCI severity groups. The distributions of the factor scores in the SCI severity groups are presented as a boxplot in Supplementary Figure 1. For Factor score 1, all pairwise comparisons were statistically significant except for the comparison between group D and group C5-C8 A, B, and C. For Factor score 2, group D differed statistically significantly from

all other groups, but the other pairwise comparisons did not exhibit statistically significant results. Wilcoxon tests were conducted to study the differences between the factor scores within each group separately. In all groups, the difference between Factor score 1 and Factor score 2 was statistically significant.

In conclusion, the factor structure of the questionnaire is quite clear: the questionnaire represents the dimensions of daily activities and mobility. Group D and group T1-S5 A, B, and C can be distinguished clearly from the other groups, but group C1-C4 A, B, and C and group C5-C8 A, B, and C seem to be harder to distinguish from one another.

References

(1) Kaiser HF. An index of factorial simplicity. Psychometrika 1974;39:32-36.

Supplementary Table B. Factor loadings of the SCIM-SR in the FinSCI study.

Variables		Factor 1 Daily activities	Factor 2 Mobility	Communalities
1	Eating and drinking	0,75	0,13	0,58
2A	Washing your upper body and head	0,74	0,38	0,70
2B	Washing your lower body	0,73	0,48	0,76
3A	Dressing your upper body	0,87	0,28	0,83
3B	Dressing your lower body	0,81	0,40	0,81
4	Grooming	0,77	0,18	0,62
5	Breathing	0,31		0,10
6	Bladder management	0,37	0,56	0,45
7	Bowel management	0,29	0,12	0,10
8	Using the toilet	0,73	0,50	0,79
9	Movement without assistance or electric aids	0,55	0,21	0,35
10	Transfers from the bed the wheelchair	0,72	0,46	0,73
11	Transfers from the wheelchair to the toilet/tub	0,68	0,54	0,75
12	Moving around indoors	0,25	0,92	0,91
13	Moving around moderate distances (10 to 100 m)	0,26	0,93	0,93
14	Moving around outdoors for more than 100 m	0,27	0,90	0,88
15	Going up or down stairs	0,24	0,89	0,85
16	Transfers from the wheelchair into the car	0,59	0,61	0,72
17	Transfers from the floor to the wheelchair	0,35	0,77	0,72
	Eigevalue	11.0	2.1	
	Explaining	57.9%	11.0%	

Extraction Method: Principal Axis Factoring.
Rotation Method: Varimax with Kaiser. Normalization.

Rotation converged in 3 iterations.

Supplement Figure A. Distributions of the factor scores across severity of SCI groups (median, percentiles 25; 75, min, max) in the FinSCI study.

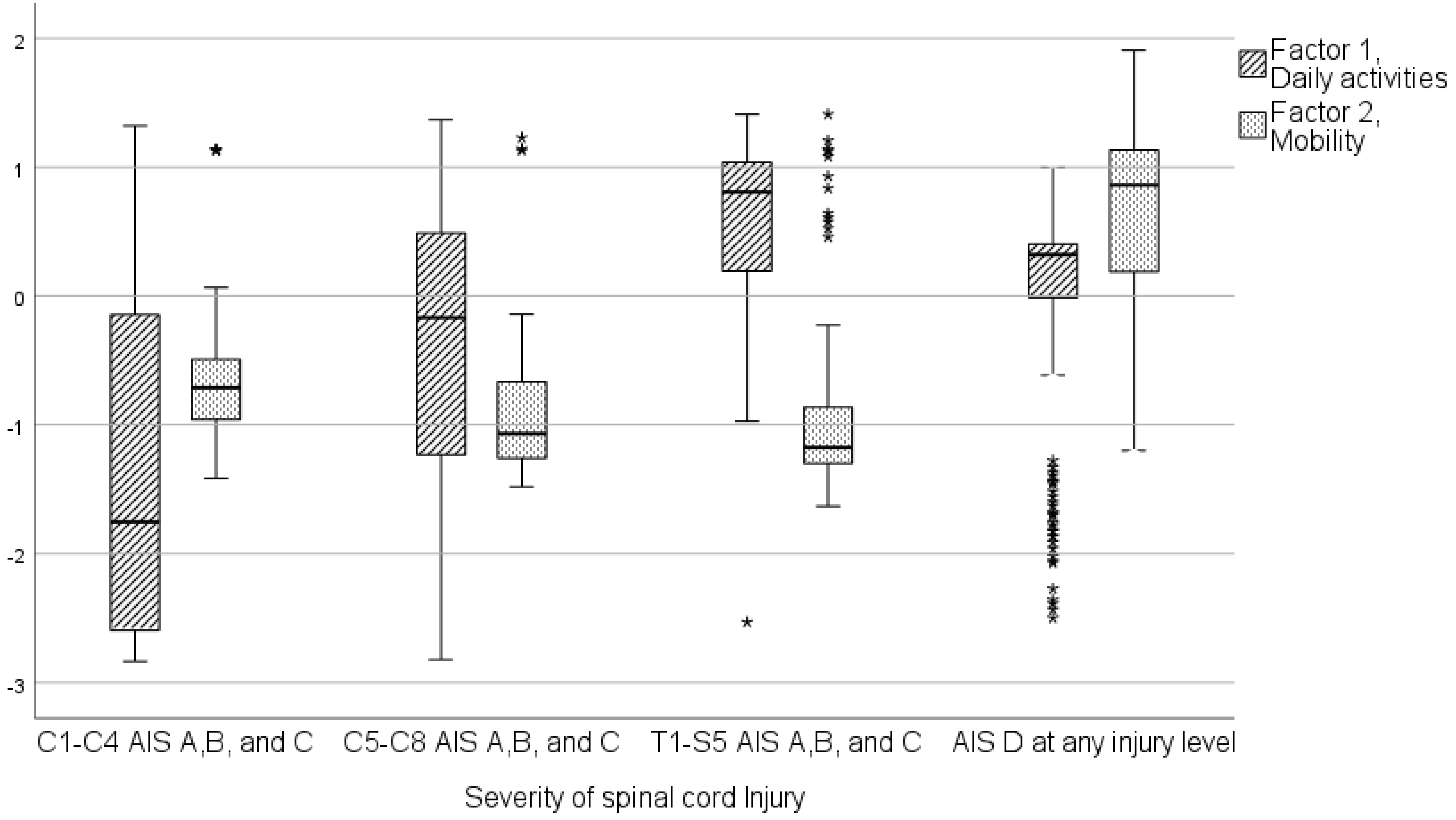

Supplement: Supplementary file 1 — Supplement [file 41393_2021_700_MOESM1_ESM.pdf]
